# Subtle Paranodal Injury Slows Impulse Conduction in a Mathematical Model of Myelinated Axons

**DOI:** 10.1371/journal.pone.0067767

**Published:** 2013-07-03

**Authors:** Charles F. Babbs, Riyi Shi

**Affiliations:** Department of Basic Medical Sciences, Center for Paralysis Research, and Weldon School of Biomedical Engineering, Purdue University, West Lafayette, Indiana, United States of America; MGH, MMS, United States of America

## Abstract

This study explores in detail the functional consequences of subtle retraction and detachment of myelin around the nodes of Ranvier following mild-to-moderate crush or stretch mediated injury. An equivalent electrical circuit model for a series of equally spaced nodes of Ranvier was created incorporating extracellular and axonal resistances, paranodal resistances, nodal capacitances, time varying sodium and potassium currents, and realistic resting and threshold membrane potentials in a myelinated axon segment of 21 successive nodes. Differential equations describing membrane potentials at each nodal region were solved numerically. Subtle injury was simulated by increasing the width of exposed nodal membrane in nodes 8 through 20 of the model. Such injury diminishes action potential amplitude and slows conduction velocity from 19.1 m/sec in the normal region to 7.8 m/sec in the crushed region. Detachment of paranodal myelin, exposing juxtaparanodal potassium channels, decreases conduction velocity further to 6.6 m/sec, an effect that is partially reversible with potassium ion channel blockade. Conduction velocity decreases as node width increases or as paranodal resistance falls. The calculated changes in conduction velocity with subtle paranodal injury agree with experimental observations. Nodes of Ranvier are highly effective but somewhat fragile devices for increasing nerve conduction velocity and decreasing reaction time in vertebrate animals. Their fundamental design limitation is that even small mechanical retractions of myelin from very narrow nodes or slight loosening of paranodal myelin, which are difficult to notice at the light microscopic level of observation, can cause large changes in myelinated nerve conduction velocity.

## Introduction

The majority of the axons in vertebrate nervous systems are wrapped with insulating layers of back-to-back cell membranes called myelin. The mechanisms by which myelin speeds axonal conduction in health and by which damage to myelin leads to loss of axonal conduction in disease have attracted much attention [1]. Still, the causes of myelin related functional deficits remain incompletely understood [2]–[4]. Continuing multidisciplinary investigation combining morphology, electrophysiology, and mathematical modeling may elucidate critical mechanisms and perhaps guide the development of effective treatments for medical conditions involving myelin damage, including traumatic injury to the brain or spinal cord and demyelinating diseases such as multiple sclerosis.

**Figure 1:**
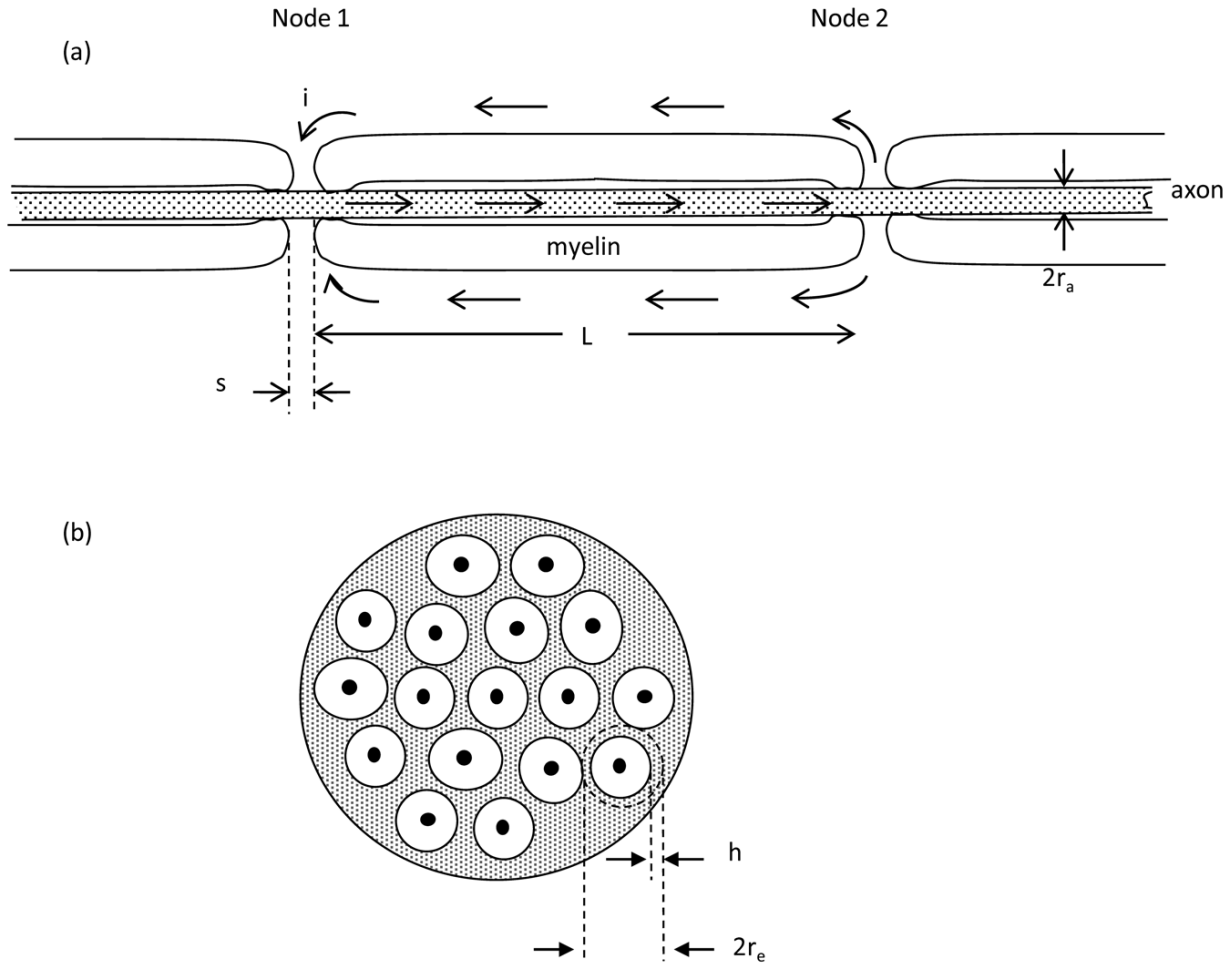
Relevant anatomy. (a) Schematic longitudinal section of a myelinated axon. The width of each node of Ranvier is denoted s. The distance between nodes is denoted L. Arrows indicate flow of positive ionic current during depolarization of Node 1 as conduction of the action potential moves toward Node 2. This sketch is foreshortened in the axial dimension. Anatomically L/s ∼ 1000. (b) Schematic cross section of a myelinated nerve or fiber tract. Each axon (solid black) is surrounded by a sheath of myelin (white) and in turn surrounded by a sheath of non-myelinated tissue (shaded) known as endoneurium or neuropil. The mean radius of the sheath of endoneurium is denoted r_e_ and the thickness of the sheath is denoted h. The cross section of endoneurium, 2πr_e_h, is much greater than that of the axon.

Myelin forming Schwann cells in the peripheral nervous system or oligodendrocytes in the central nervous system wrap around the axon multiple times to create laminated layers of insulating cell membrane, as shown schematically in Figure 1. Periodic short gaps in the myelin sheath along the axons having width, s, approximately 0.3 to 1 micrometer, are the Nodes of Ranvier, where the density of transmembrane channels carrying inward sodium current is high and where transmembrane action potentials are initiated. Immediately adjacent to the nodes themselves on either side in the axial dimension are the paranodal regions, where myelin is tightly attached to the underlying axonal membrane. Beyond the paranodal regions are the functionally important juxtaparanodal zones, where the density of transmembrane channels carrying outward potassium current is high. Ring-like bands of paranodal cell-cell attachments separate and insulate the bare nodes from the juxtaparanodal regions. Since myelin inhibits the conduction of ionic current, the action potential tends to jump from one node to the next along the longitudinal axis of an axon. This process of jumping, or “saltatory conduction”, boosts the speed of propagation of action potentials along myelinated axons to tens of meters per second, rather than tens of centimeters per second typical of unmyelinated axons [5].

Here we explore in detail the functional consequences of subtle injury to the well-known anatomic arrangement of the nodes of Ranvier, coupled with the more recently discovered segregation of nodal sodium channels from juxtaparanodal potassium channels [6]–[8]. Compared to the internodal regions, a node of Ranvier has a relatively high density of sodium channels, which enable the generation of action potential at the node. The virtual absence of sodium channels in the paranodal and juxtaparanodal regions, together with high electrical resistance of the paranodal region, blocks axial ionic current beneath the myelin sheath and favors saltatory conduction. Potassium channels, on the other hand, are segregated from sodium channels in nodes of Ranvier, residing predominantly beneath the myelin at the juxtaparanodal area, and separated from the node by the paranodal region [9]. There is now general agreement that mechanical crush or stretch injury results in myelin damage, especially in the paranodal region [10], [11]. This idea is consistent with biomechanical modeling showing significant stress in this area under mechanical insults [12], causing retraction of myelin away from the node [13], [14]. With more severe injury there is also de-coupling and detachment of paranodal myelin, opening a low resistance gap or sleeve-like channel between the node and the juxtaparanodal region, through which increased potassium ion current may flow [10].

Subtle retraction and detachment of paranodal myelin can have two electrical effects. The first is increasing bare nodal area, which increases the electrical capacitance of the node. The second is loosening of paranodal cell-cell junctions, which reduces the normally high electrical resistance between the juxtaparanodal potassium channels and the nodal capacitance. In the present paper we consider the biophysics of nodal function before and after traumatic myelin retraction and partial detachment to explain and predict the effects of such subtle mechanical injury, which is sufficiently mild that it is clearly visible only at the electron microscopic level of observation [10]. Our approach is to employ a mathematical model of ionic current flow that reflects changes caused by such subtle injury to the nodal and paranodal regions.

The use of mathematical models to study mechanisms of saltatory conduction is well precedented [15]–[24]. Classical and more modern approaches almost universally involve a form of cable equation or cable model, as originally described by FitzHugh [15]. This approach regards successive nodes as a chain of leaky capacitors having voltage sensitive ion channels, and connected in parallel by extracellular and intracellular resistances between the nodes. Currents and voltages at each node are computed from a second order partial differential equation that describes the voltages at each node as functions of time and space. Such equations are known as cable equations, because of their similarity to cable or transmission line equations [25], and the corresponding models of nerve conduction are known as cable models. Subsequent, more detailed mathematical treatment of composite, myelinated axon models [23], [24] leads to expressions essentially similar to the original solutions of FitzHugh. (Compare, for example, Basser Eq. (35) and Nygren and Halter Eq. (18) with FitzHugh Eq. (1)).

The goal of the present paper is to determine quantitatively how myelinated nerve conduction velocity depends upon the local integrity of the nodes and paranodal regions at the electron microscopic level of observation and how nerve conduction might be slowed or blocked by the known pathological changes to these structures [2]. We also explore how drug treatments that block potassium conductance in the juxtaparanodal region may act to restore conduction in subtly damaged regions. Toward this end it is insightful to derive from first principles a FitzHugh style cable model of myelinated axon conduction for the specific purpose of characterizing the parameters related to subtle injury.

## Methods

### Model of a Myelinated Axon

**Figure 2:**
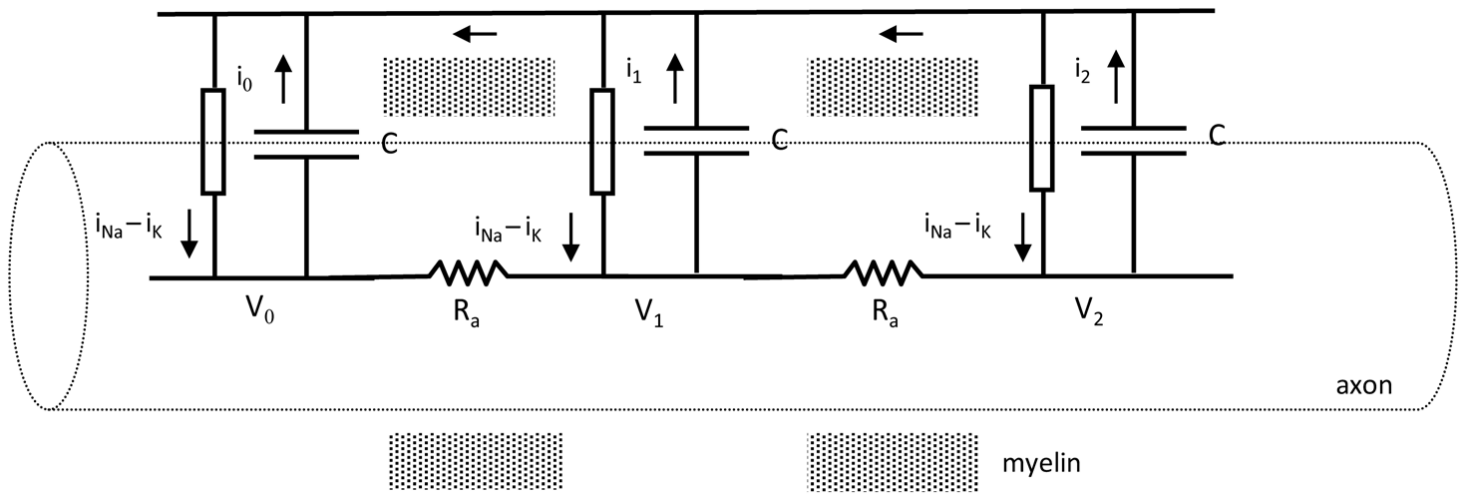
Electrical model for multiple nodes of Ranvier. Arrows indicate directions of positive ionic current. Shading indicates foreshortened myelinated regions. In life the actual distance between nodes (∼1000 µm) is much greater than the width of a single node (∼1 µm). Current is denoted by i, capacitance by C, resistance by R, and voltage by V. Lumped ionic currents from sodium and potassium channels in and around each node are shown as a single current source.

A simplified equivalent electrical circuit for a series of equally spaced nodes of Ranvier, together with associated paranodal and juxtaparanodal regions, is shown in Figure 2. The intracellular resistance between nodes along the axons is denoted R_a_ and is much larger than electrical resistance of the extracellular current path between nodes, R_e_, (not show in Figure 2). The nodal capacitances of the exposed, non-myelinated axon segments at each node are represented as 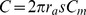, where C_m_ is the axon membrane capacitance per unit area, s is the span of bare, unmyelinated axon in a node of Ranvier, and r_a_ is the radius of the axon. Definitions of symbols and variables are provided for reference in Table 1.

**Table 1:** Nomenclature.

Symbol	Definition	Units
A	Area	cm^2^
C	Capacitance of bare membrane in a node of Ranvier	farads
C_m_	Specific membrane capacitance per unit area	farads/cm^2^
d	Axon diameter	cm
e	Base of natural logarithms (2.718…)	
G	Membrane conductance per unit area	ohm^−1^ cm^−2^
h	Thickness of a conducting sheath surrounding a myelinated axon	cm
i, i_0_, i_1_	Positive ionic current during an action potential	amperes
λ	Length of juxtaparanodal region	cm
L	Distance between nodes of Ranvier	cm
n	Number of nodes in a discrete model	
π	Circle ratio (3.1415 …)	
R_a_	Axonal electrical resistance between nodes of Ranvier	ohms
R_e_	Extracellular electrical resistance between nodes of Ranvier	ohms
R_p_	Paranodal electrical resistance between a node and both juxtaparanodal regions of a myelinated axon	ohms
r_a_	Radius of an axon	cm
r_e_	Radius of a conducting sheath surrounding a myelinated axon	cm
ρ, ρ_e_, ρ_a_	Resistivity of extracellular or axonal fluid	ohm-cm
s	Span of bare axon in a node of Ranvier in the axial dimension	cm
t	Clock time	sec
τ	Clock time of activation of a node	sec
t′	Time after node activation (t − τ)	sec
V	Transmembrane potential difference	volts
V_th_	Threshold potential for initiation of an action potential	volts
v	Nerve conduction velocity	m/sec

Because of the relatively large distance between nodes compared to the node width, the segregation of ion channels for inward sodium current and outward potassium current in the nodal and juxtaparanodal regions does not change or invalidate the simplified equivalent circuit shown in Figure 2 as a realistic, model of a myelinated axon segment. However, the potassium current sources in the juxtaparanodal regions are a few micrometers distant from the sodium current sources in the bare nodal regions. Potassium current must flow between the axolemma and tightly adherent myelin in the paranodal regions in order to repolarize the nodal capacitance. This ultrastructural detail may add a substantial series resistance to the potassium ion “battery”, which is omitted for simplicity in Figure 2 but described in detail in Appendix S1.

To specify extracellular and axonal resistances, R_e_ and R_a_, we note that the resistance of a volume conductor in terms of its length, cross-section, and resistivity is
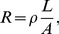
(1a)where ρ is resistivity, L is length, and A is cross sectional area [26]. Resistivity is the intrinsic property of a material that opposes the flow of steady electric current and is expressed in units of ohm-cm. From the geometry of Figure 1 we can write algebraic expressions for the internal resistance of an axon, R_a_, between nodes as a function of the axon radius, r_a_, and also for the external resistance, R_e_, of the sleeve of tissue surrounding the myelin, through which current must flow between two adjacent nodes to complete the electrical circuit. For R_a_ and R_e_ we have
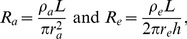
(1b)where a sleeve of aqueous extracellular fluid of thickness h and mean radius re surrounds the myelin sheath over distance L and has extracellular fluid resistivity, ρe. Noting that extracellular resistance is about two orders of magnitude less than axonal resistance leads to a compact model shown in Figure 2.

### Time-varying Sodium and Potassium Currents

Local inward sodium currents, i_Na0_, i_Na1_, i_Na2_, … and outward potassium currents i_K0_, i_K1_, i_K2_, … for nodes 0, 1, 2, … and nearby juxtaparanodal regions are governed by time varying transmembrane conductances, G_Na0_, G_Na1_, G_Na2_ … for sodium and G_K0_, G_K1_, G_K2_ …. for potassium and the respective sodium and potassium equilibrium potentials [5]. Convenient descriptive formulas for ion specific conductances as functions of time, 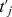, after onset of activation of node, j, (including both the bare nodal membrane and functional juxtaparanodal regions) have the form:
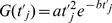
(2)for constants, a and b.

Such functions nicely describe the classical waveforms of time-varying conductance determined experimentally in units of Seimens per square centimeter. It is easy to show using calculus that the values of a and b required to produce a peak conductance G* at time t* after onset of activation are given by the expressions 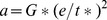 and 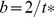, were e is the base of the natural logarithms ≈2.781. In turn, values of a and b describing textbook normal activation functions for sodium and potassium conductance [5] can be computed as shown in Table 2. Specifically, we assume for the purpose of this study that the densities of sodium and potassium of ion channels per square centimeter of membrane in the regions of the nodes of Ranvier where such channels are concentrated is similar to that classically described for non-myelinated axons [6].

**Table 2:** Constants for descriptive functions for membrane conductance per unit area, 
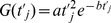
.

	Coordinates of peak conductance [5]		Model function constants	
	t* (sec)	G* (ohm^–1^cm^−2^)	a (ohm^–1^cm^–2^sec^–2^)	b (sec^–1^)
Na^+^	0.0001	0.028	2.1×10^7^	2×10^4^
K^+^	0.0005	0.013	3.8×10^5^	4×10^3^

Using these descriptive functions for time-dependent sodium and potassium conductance per square centimeter of membrane area, the corresponding local nodal currents at node, j, and adjacent paranodal and juxtaparanodal membranes of the model become
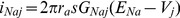
(3a)and
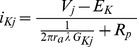
(3b)in terms of the axon radius, ra, unstretched node width, s, length of juxtaparanodal region on both sides of the node, λ, local prevailing transmembrane potential, Vj, and the sodium and potassium equilibrium potentials ENa and EK, as described in [5]. In calculating potassium current, the juxtaparanodal length, λ, including both sides of the node, is taken as approximately 10 times the normal nodal length based on cytochemical studies of potassium ion distribution [4], [14]. Expression (3b) represents the current passing through the juxtaparanodal membrane conductance in series with paranodal resistance, Rp. Rp is the resistance of the thin sleeve of extracellular matrix material through which potassium current must flow from the juxtaparanodal regions to the bare axonal membrane of the local node of Ranvier. Note for the case Rp = 0 the expression for potassium current is analogous to that for sodium current. If Rp were exceedingly large, then potassium current would be zero. Details of the treatment of potassium current in the presence of paranodal resistance and the estimation of a numerical value for paranodal resistance are provided in Appendix S1.

The same conductance functions G_Na_(t′_j_) and G_K_(t′_j_) are used for all nodes. However, the time variable, t′_j_, for each node differs among nodes, depending on the time of activation. Here we let τ_j_ represent the clock time of activation of nodes j = 0, 1, 2, … n in the model. In particular, time τ_j_ is defined as the clock time, t, when the transmembrane potential V_j_(t) equals or exceeds the threshold potential V_th_ for each node j = 0, 1, 2, …. In turn, t′_j_ = max (0, t−τ_j_). Then the node-specific ionic conductance is computed for times t>τ_j_ as 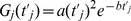. For Node 0, the first node in the chain, τ_0_ = 0, so that activation begins at node 0, proceeding from left to right in Figure 2.

### Solving for Membrane Potentials at Successive Nodes

The model of the myelinated axon includes an arbitrary number ∼20 nodal capacitances, connected by equal axonal resistances, 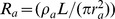, through which ionic current may flow, as shown in Figure 2. The capacitances, 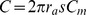, correspond to the bare membrane areas of successive nodes of Ranvier. Transmembrane potential is specified as the intracellular minus extracellular electrical potential at each node. To simulate myelinated nerve conduction node 0 at the left boundary is actively depolarized by a suprathreshold stimulus. The successive nodes 1, 2, … n become active if their membrane potential exceeds a threshold value, V_th_, such as –50 mV.

The computational approach used here is an adaptation of that previously published for modeling the circulatory system by one of us [27], based upon the definition of capacitance and Ohm’s Law. The definition of capacitance is 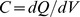, where C is nodal capacitance and 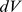 is the incremental change in electrical potential or voltage across the capacitance as charge 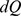 is introduced. For current i = dQ/dt flowing during time increment, dt, we must have dV/dt = i/C. Ohm’s Law, which relates current to voltage and resistance, is 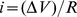, where 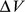 is the instantaneous difference in voltage across resistance R as current i occurs.

Applying these basic concepts with reference to Figure 2 provides a set of governing differential equations that can be used to describe transmembrane potentials in the chain of nodes. Beginning with the most distant node, n, we set as a boundary condition
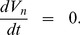
(4)


For active nodes the net current flowing onto a nodal capacitance is the difference between inflow to and outflow from the node. Thus, the general structure of equations (5) and (6) below is: rate of voltage change = transmembrane sodium current inflow, minus transmembrane potassium current outflow, plus axon current inflow, minus axon current outflow, all divided by local capacitance. Ohm’s Law allows calculation of axon current inflow and outflow from voltages at adjacent nodes. For interior nodes j = 1, 2, … n –1
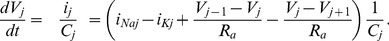
(5)


For left hand node 0
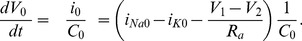
(6)


### Estimation of other Parameters

We consider a textbook normal case as a primary model. Membrane ionic conductances per square centimeter are as listed in Table 2. From anatomic sections such as those in [28] the radius of the sleeve of fibrous tissue is about 6 micrometers and the thickness of the sleeve is about 2 micrometers. Axon diameter averages 1.0 micrometer [29]–[31]. The width, s, of a normal node of Ranvier ranges from about 0.3 micrometer to about 1.0 micrometer, or 0.3 to 1.0 times the diameter of an axon, in various anatomic specimens [28]. A middle value for the width, s, of a node is about 0.65 micrometer [10], [32]. The span of the juxtaparanodal region, λ, on both sides of a normal node is taken as 10 times the axon radius [33]. The resistivity of intracellular fluid is about 200 ohm-cm [31]. The paranodal resistance, R_p_, is estimated as 3.2×10^10^ ohms, as explained in Appendix S1. The specific membrane capacitance of nerve cell membranes is about 1 mircofarad per square centimeter [5]. Using these values to evaluate parameters, together with normal membrane potentials [5] (resting membrane potential –85 mV, threshold potential –50 mV, sodium equilibrium potential +67 mV, potassium equilibrium potential –95 mV), one can specify the values of all parameters for a standard normal model.

### Numerical Integration

To describe waveforms of membrane potential vs. time at each node j = 0, 1, 2, … n –1 equations (5) and (6) for dV/dt were integrated numerically using the simple Euler method, implemented in Visual Basic code within an Excel spreadsheet on an ordinary personal computer. For each node the membrane potential V_j_ at successive time steps Δt was calculated as
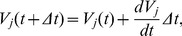
(7)using expressions (4), (5), and (6) for dV_j_/dt. Initial conditions at t = 0 describe the state of the axon segment at rest, with V_j_ set to the resting transmembrane potential for j = 1, 2, … n. Only node 0 is active at time zero (τ_0_ = 0). Nodes j = 1, 2, … n –1 become active when t>τ_j_. As a boundary condition, the rightmost node, n, in the chain is clamped at the resting potential and is never activated.

A model of 21 nodes (including bare nodal axon and functional juxtaparanodal membrane) separated by a nominal average internodal distance, L, of 1 mm represented a hypothetical axon segment 2 cm in length. The combined bare nodal, paranodal, and juxtaparanodal regions of each node had a total axial length on the order of 0.01 mm or about 1 percent of the axial distance between nodes. Given initial conditions at t = 0, the evolution of potentials V_j_(t) was computed as a “marching solution”, for which stability and accuracy are ensured by using a sufficiently small value of Δt, such as 0.1 microsecond. Increasing or decreasing Δt without effect on the results confirmed that a sufficiently small value was chosen for Δt. Wave propagation typically stabilized over three successive nodes.

### Simulation of Injury and Drug Treatment

In models of localized crush injury of the spinal cord an impulse was initiated at node 0. Nodes 1 through 7 had normal parameters, representing undamaged tissue. A mild form of stretch or crush injury causing retraction of myelin around stretched nodes was simulated by increasing the width of exposed nodal membrane, s, in nodes 8 through 20. Normal nerve conduction velocity was taken as the inter-nodal distance, divided by the steady-state wave propagation time between nodes 4 and 5, namely (τ_5_–τ_4_)/L. Injured nerve conduction velocity was taken as the inter-nodal distance, divided by the steady-state wave propagation time between nodes 15 and 16, namely (τ_16_–τ_15_)/L. In some simulations a more severe form of stretch or crush injury was simulated that included both retraction and detachment of paranodal myelin. Paranodal myelin detachment, that is, radial separation of paranodal myelin from the underlying axon membrane, was simulated by decreasing paranodal resistance to one tenth or one hundredth of its normal value. In other simulations the effect of the potassium channel blocking drug, 4-aminopyridine, was simulated by decreasing peak potassium conductance to 20 percent of its normal value, representing mild-to-moderate inhibition that would be reasonable to achieve experimentally.

## Results

### Normal Nerve Conduction

**Figure 3:**
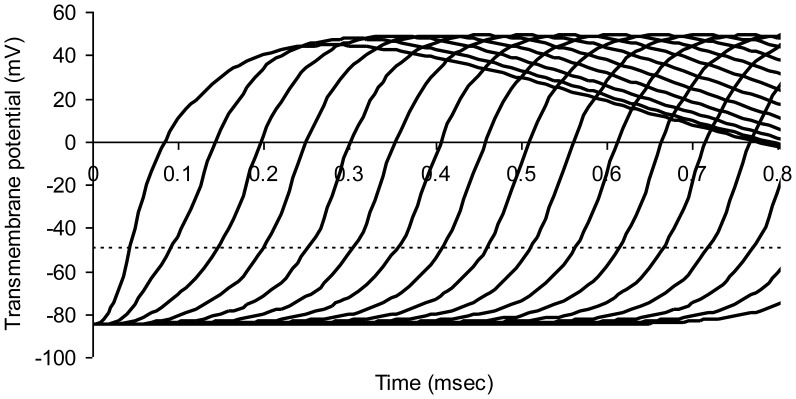
Modeled action potentials at successive nodes of Ranvier in a normal myelinated axon. The dotted horizontal line represents threshold potential. Average axon diameter 1.0 micrometer; number of nodes of Ranvier 21; node width 0.65 micrometer; width of juxtaparanodal region on both sides of a node 5.0 micrometers. The resistivity of intracellular fluid 200 ohm-cm; normal paranodal resistance 3.2×10^10^ ohms; specific membrane capacitance of axonal membrane 1 mircofarad per square centimeter; resting axonal membrane potential –85 mV; threshold potential –50 mV; sodium equilibrium potential +67 mV; potassium equilibrium potential –95 mV. The time step for numerical integration was 0.1 microsecond.

Figure 3 shows successive action potentials in a normal myelinated axon model. Transmembrane potentials for nodes numbered 0 through 16 in the series are shown in successive curves from left to right. A propagated wave of excitation travels down the chain of nodes from left to right. Nerve conduction velocity is 19.1 m/sec.

**Figure 4:**
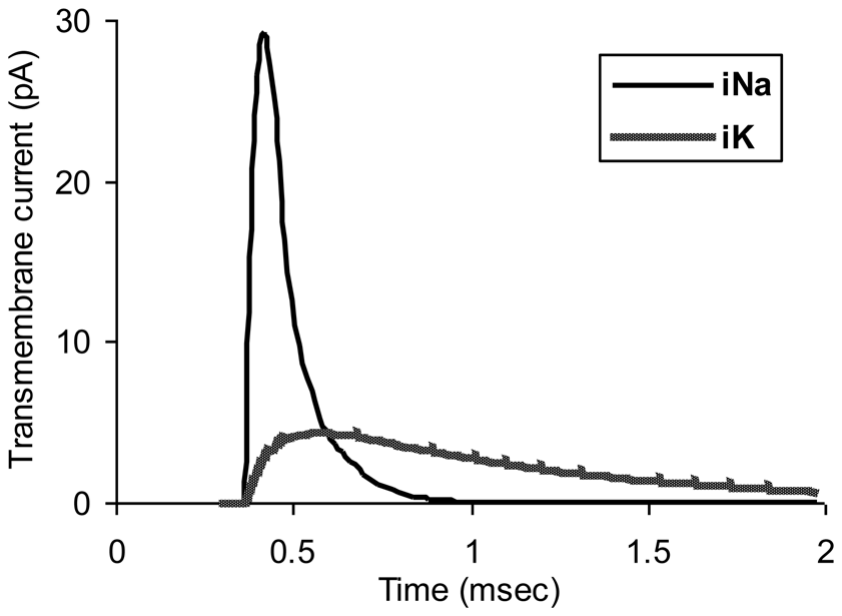
Normal ionic currents for node 6 in the myelinated axon model of Figure 3.

Figure 4 shows transmembrane sodium and potassium currents in this normal model as a function of time for node number 6 in the chain of normal nodes. The peak of potassium conductance is blunted somewhat, compared to that in an unmyelinated axon, because of the effect of paranodal resistance, R_p_, the anatomic substrate for which is the thin sleeve of extracellular matrix material and tight junctions in the paranodal region between the axolemma and overlying myelin. Recharge current for the node must pass through this normally thin space to reach the nodal capacitance.

**Figure 5:**
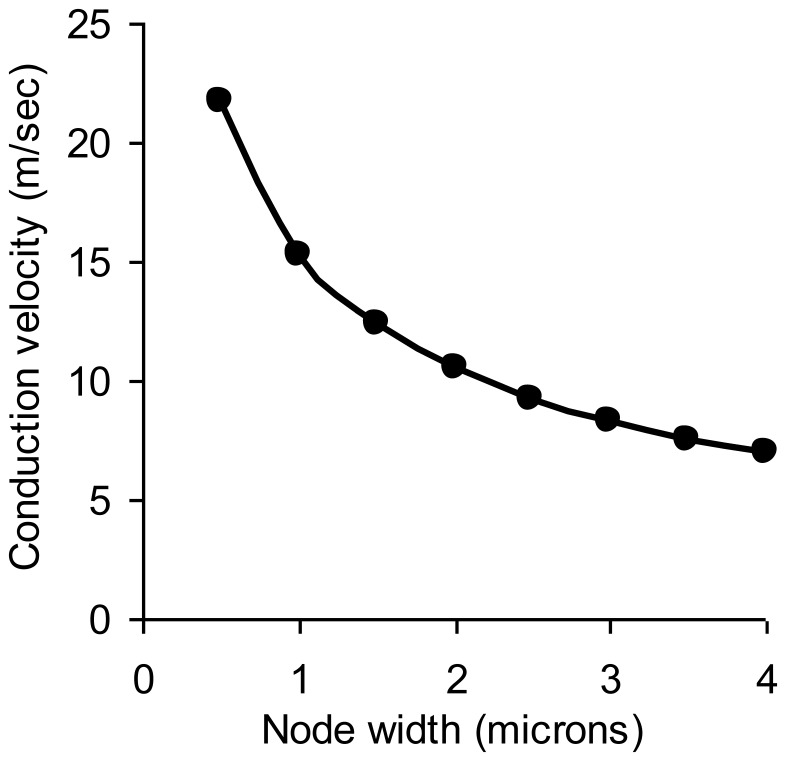
Normal myelinated nerve conduction velocity as a function of the node width.

Conduction velocity in otherwise normal myelinated axons depends on node width, s, measured along the axial dimension, as shown in Figure 5.

### Simulated Crush Injury

**Figure 6:**
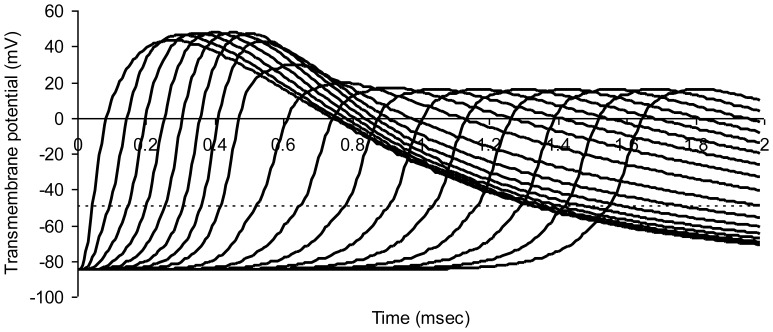
Modeled action potentials at successive nodes of Ranvier in a model myelinated axon. Simulated crush injury to nodes on the right. The dotted horizontal line represents threshold potential. Average axon diameter 1.0 micrometer; number of nodes of Ranvier 21; normal node width 0.65 micrometer; injured node width 1.95 micrometer; width of juxtaparanodal region on both sides of a node 5.0 micrometers. The resistivity of intracellular fluid 200 ohm-cm; normal paranodal resistance 3.2×10^10^ ohms throughout the model; specific membrane capacitance of axonal membrane 1 mircofarad per square centimeter; resting axonal membrane potential –85 mV; threshold potential –50 mV; sodium equilibrium potential +67 mV; potassium equilibrium potential –95 mV. The time step for numerical integration was 0.1 microsecond.

Figure 6 illustrates axonal conduction in a model of simulated crush injury. The widths, s, of nodes 8 through 20 on the right have been stretched from 0.65 µm to 1.95 µm, simulating the degree of nodal stretching observed in experimental crush injury [10]. However, the length of axon over which sodium channels are concentrated, s*, is not changed in keeping with the pre-injury segregation of sodium channels in the nodal region. Here s* is substituted for s in Equation (3a). In the crushed segment (right) the time between action potentials at successive nodes increases, indicating slowed impulse conduction.

In this example crush injury diminishes action potential amplitude by about one third and also diminishes the slope of the rising phase of the action potential, slowing conduction velocity from 18.8 m/sec in the normal region on the left to 7.8 m/sec in the crushed region on the right. The longer discharge time required for stretched nodes allows for greater outward potassium current to develop (Figure 4), and in turn less net inward positive current inflow during the rising phase of the action potential.

The reduced action potential amplitude after stretch injury appears to happen for the following reason. The currently depolarizing node of Ranvier acts as a constant current source in the presence of stretch injury. When mild stretch injury increases node width, the length of axon over which sodium channels are concentrated is not changed, in keeping with pre-injury segregation of sodium channels in the nodal region. However, simple stretch injury increases the nodal surface area, and in turn increases the nodal capacitance. When the increased nodal capacitance of the next downstream node is discharged by a constant current source the rate of change in voltage is reduced (dV/dt = i/C, where current, i, is constant and capacitance, C, is increased). The reduced slope between the threshold potential and the peak of the action potential reflects the increased capacitance. The peak does not reach normal height, because a reduced slope over a constant duration results in a reduced peak amplitude.

The predicted results are similar to the diminished amplitude and increased latency of compound action potentials observed experimentally by Shi and Blighty [34]. Because node width, s, is on the order of only one micrometer normally, subtle damage, separation, or retraction of the myelin sheath at the nodes of Ranvier might go unnoticed at the light microscopic level of observation. Such damage, however, can lead to substantial degradation in myelinated nerve conduction.

**Figure 7:**
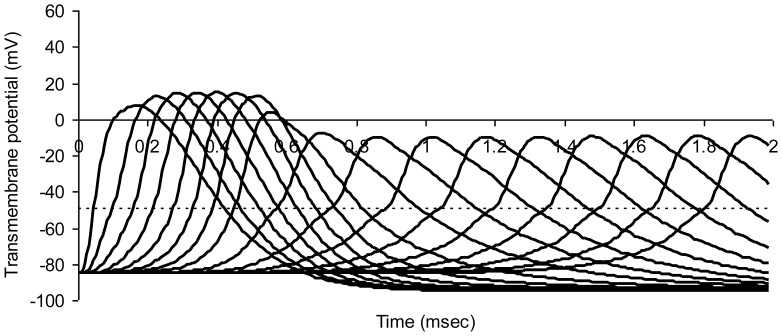
Modeled action potentials at successive nodes of Ranvier in a model myelinated axon. Simulated crush injury to nodes on the right. The dotted horizontal line represents threshold potential. In this simulation node width was increased three-fold and paranodal resistance was decreased to one tenth the normal value. Average axon diameter 1.0 micrometer; number of nodes of Ranvier 21; normal node width 0.65 micrometer; injured node width 1.95 micrometer; width of juxtaparanodal region on both sides of a node 5.0 micrometers. The resistivity of intracellular fluid 200 ohm-cm; normal paranodal resistance 3.2×10^10^; injured paranodal resistance 3.2×10^9^ ohms; specific membrane capacitance of axonal membrane 1 mircofarad per square centimeter; resting axonal membrane potential –85 mV; threshold potential –50 mV; sodium equilibrium potential +67 mV; potassium equilibrium potential –95 mV. The time step for numerical integration was 0.1 microsecond.

When decreased paranodal resistance is included in the model, such as would occur with decompaction or detachment of paranodal myelin [10], conduction is further degraded (Figure 7). When node width is stretched three-fold (similar to that observed in [10]), and paranodal resistance is reduced to one-tenth normal, the nerve conduction velocity is decreased from 17 m/sec in the uninjured region on the left to 6.6 m/sec in the injured region on the right. The amplitude of the action potential is also further reduced. Evidently, decreased paranodal resistance allows more outward potassium current to reach the node, diminishing the net depolarizing current, the rate of rise of the action potential, and the peak amplitude of the action potential. Note that the 90 percent reduction in R_p_ represents very subtle injury in this model since a retraction of the paranodal junction leaving a gap of only 0.1 micron around the circumference of the axon would reduce R_p_ by >99.9 percent.

**Figure 8:**
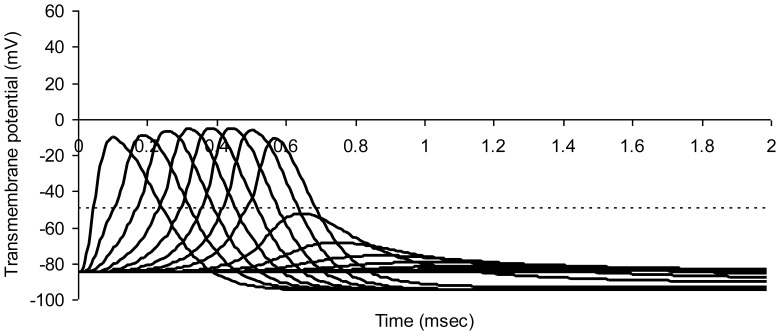
Modeled action potentials at successive nodes of Ranvier in a model myelinated axon. Simulated crush injury to nodes on the right with 300 percent increase in bare nodal width and a 99% decrease in paranodal resistance. The dotted horizontal line represents threshold potential. Average axon diameter 1.0 micrometer; number of nodes of Ranvier 21; normal node width 0.65 micrometer; injured node width 1.95 micrometer; width of juxtaparanodal region on both sides of a node 5.0 micrometers. The resistivity of intracellular fluid 200 ohm-cm; normal paranodal resistance 3.2×10^10^; injured paranodal resistance 3.2×10^8^ ohms; specific membrane capacitance of axonal membrane 1 mircofarad per square centimeter; resting axonal membrane potential –85 mV; threshold potential –50 mV; sodium equilibrium potential +67 mV; potassium equilibrium potential –95 mV. The time step for numerical integration was 0.1 microsecond.

When paranodal resistance if further reduced to 1 percent of the normal value, together with a three-fold increase in bare nodal width, there is complete conduction block in the region of simulated injury (Figure 8).

**Figure 9:**
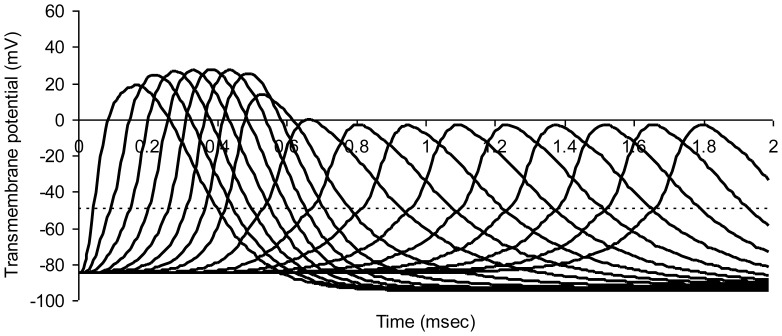
Treatment of simulated crush injury to nodes on the right with a potassium channel blocker that reduces peak potassium conductance in all nodes by 80%. Parameters of injury: 300 percent increase in bare nodal width and a 99% decrease in paranodal resistance. The dotted horizontal line represents threshold potential. Average axon diameter 1.0 micrometer; number of nodes of Ranvier 21; normal node width 0.65 micrometer; injured node width 1.95 micrometer; width of juxtaparanodal region on both sides of a node 5.0 micrometers. The resistivity of intracellular fluid 200 ohm-cm; normal paranodal resistance 3.2×10^10^; injured paranodal resistance 3.2×10^8^ ohms; specific membrane capacitance of axonal membrane 1 mircofarad per square centimeter; resting axonal membrane potential –85 mV; threshold potential –50 mV; sodium equilibrium potential +67 mV; potassium equilibrium potential –95 mV. The time step for numerical integration was 0.1 microsecond.

Blocked conduction in the simulation of Figure 8 can be restored by inhibiting peak potassium conductance in all nodes of the model by 80%, that is, by replacing G_Kmax_ = 0.013 S/cm^2^ with introducing G_Kmax_ = 0.2*0.013 S/cm^2^. The effect of simulated drug treatment with a potassium channel blocker is shown in Figure 9. Conduction is restored. However, conduction velocity remains low at 7.1 m/sec.

## Discussion

Mathematical modeling can help to organize and distill knowledge about complex systems, highlight the most important variables that govern system performance, and suggest testable hypotheses for future research. The simple computational model of myelinated nerve conduction presented here provides several insights into the normal functioning and the failure of myelinated axons, which remain points of discussion in the 21^st^ century [35], [36]. One critical and underappreciated variable is the width of the nodes themselves, compared to the diameter of the axon. Another is the paranodal electrical resistance that separates the high density of sodium channels in the node itself from the juxtaparanodal potassium channels.

Narrower nodes increase conduction velocity (Figure 5) as previously pointed out in a semi-quantitative way by Giuliodori and DiCarlo [36]. The discharge time constant, R_a_C, which governs the time required for downstream nodes to be depolarized from their resting membrane potential to the threshold potential, is simply
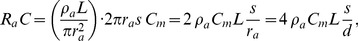
(8)where s/d is the nodal ratio or node width divided by axon diameter. This expression includes relatively few variables and holds true as long as paranodal myelin remains tightly coupled to the underlying axonal membrane, so that paranodal resistance remains normal.

The relationship of the discharge time constant (8) to nerve conduction velocity can be appreciated as follows. Consider myelinated nerve conduction velocity as the distance, L, between nodes, divided by the time, Δt, it takes for the next downstream node (e.g. Node 2 in Figure 1) to reach threshold after the transmembrane potential at a given node (Node 1 in Figure 1) reaches threshold and nodal sodium channels are fully open. (The value of Δt can be appreciated graphically with reference to Figure 3.) As soon as Node 1 becomes a low resistance pathway for ionic current, downstream Node 2 will depolarize to a value of about 1/e (37%) of its resting negative membrane potential in one time constant R_a_C. The time required to reach threshold is therefore 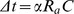, where α is a constant near 0.5. In turn, myelinated nerve conduction velocity
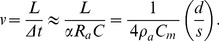
(9)


As node width, s, decreases, the myelinated nerve conduction velocity increases hyperbolically. Further, the node width and axon diameter are the major anatomic determinants of myelinated nerve conduction velocity. The direct dependence of conduction velocity on axon diameter is well established [16], [18]. However, the importance of the node width or the nodal ratio is much less well appreciated.

The sensitive dependence of myelinated nerve conduction velocity on the nodal width, s, of exposed axon membrane (or the ratio of nodal width to axon diameter, s/d) has important biological implications. Interestingly, normal nerve conduction velocity is located near the elbow of the curve in Figure 5. This subtle feature hints that mammals are a highly evolved order of vertebrates. Natural selection would likely favor animals with higher nerve conduction velocity, quicker reaction times, and quicker thinking. However, as node spacing gets closer and closer, problems could arise with quality control in forming nodes to such close tolerances. For very small values of node span, s, small absolute change in the separation of myelinated segments on either side would cause a relatively large change in s and in the nodal ratio. (Indeed, the magnitude of the derivative dv/ds is proportional to 1/s^2^.) Simple movement of peripheral nerves or very mild concussions in the brain could cause relatively large changes in nerve conduction velocity, v, and hence in the stability and predictability of the nervous system. Coordinated complex movements would become more difficult if conduction time from brain to muscle, or within the central nervous system, varied unpredictably. Very narrow nodes would be fast, but they would also be delicate and perhaps too noisy. In this way the net survival advantages of quickness might plateau or even degrade as a function of decreasing node width. We may have reached a near optimal compromise through natural selection.

The model also provides insights into the pathophysiology of crush injury. The subtle injury involving retraction and fraying of paranodal myelin increases the effective capacitance of the nodal membrane by perhaps an order of magnitude or more and also exposes paranodal and juxtaparanodal regions. Detachment of paranodal myelin, perhaps only visible at the electron microscopic level of observation [10], reduces by perhaps an order of magnitude or more the paranodal resistance through which repolarizing potassium current must flow to restore the resting membrane potential after an action potential. These small anatomical injuries increase early outward potassium current, opposing inward sodium current at the node. As a result, the time required to discharge the next downstream node of Ranvier to the threshold level is increased. In turn, nerve conduction velocity falls, since it is related to the inter-nodal distance, divided by the discharge time.

The calculated changes in conduction velocity with subtle paranodal injury in the present paper agree with experimental observations. Experiments in isolated guinea pig spinal cord by Shi and Pryor [37] revealed that the peak latency of action potentials increased by 35 percent after stretching guinea pig spinal cord segments. The stretched region in these preparations was about one third of the total distance over which latency was computed. The modified cable model derived here predicts that myelinated nerve conduction latency at node 10 in the model of Figure 7 would increase from a normal value of 0.57 sec (Figure 3) to 0.80 sec (Figure 7) a 40 percent increase in latency when the path from node 0 to node 10 in the model includes three injured nodes (only one third of the total). Shi and Prior also found that superfusion with 100 micromolar 4-aminopyridine (a potassium channel blocker) partially restored the loss in amplitude of the compound action potential 30 min after stretch injury. However, there was no significant change in conduction velocity following 4-aminopyridine treatment. Since the compound action potential represents a sum of signals from many axons, these experimental results are consistent with the model results in Figure 9, showing that an 80% blockade of potassium conductance restores conduction with slow velocity following severe stretch injury. The modeling results are also consistent with experimental studies in other animal models and with preliminary clinical trials of 4-aminopyridine as a therapeutic agent in spinal cord injury [4].

Retraction of myelin around nodes of Ranvier can be produced by chemical injury as well as by stretch injury. Fu and coworkers [14], using CARS imaging, reported paranodal myelin splitting and retraction in response to glutamate excitotoxicity in isolated rat spinal cord. The nodal ratio in these animals increased from a normal value near 1.0 to a pathological value near 3.0 after glutamate. Similarly, Shi and coworkers [32] found that nodal ratios increased from a control mean of 0.6 to 2.9 twelve hours after application of 500 micromolar acrolein to isolated guinea pig spinal cord. Subtle nodal injury, accompanied by slowed conduction velocity, is also recognized as an important mechanism in the pathology of multiple sclerosis [3], [38]. Hence the mechanisms described in this paper may have relevance beyond the field of neurotrauma.

The modeling approach used in the present study to explore the particular effects of node width and paranodal resistance builds on a rich intellectual history. The original model of saltatory conduction in myelinated nerve fibers was done by Richard FitzHugh [15]. This approach modeled nodes as strings of leaky capacitors having Hodgkin-Huxley type ion channels, and connected in parallel by extracellular and intracellular internodal resistances. Currents and voltages at each node are computed. FitzHugh derived a second order partial differential equation for this system to describe voltages at each node as functions of time and space, and solved the equation numerically. (Much later Basser [23] and Nygren and Halter [24] used analytical methods to derive equivalent equations for a composite, myelinated axon, obtaining expressions essentially isomorphic with the original solutions of FitzHugh (compare Basser Eq. 35 and Nygren and Halter Eq. 18 with FitzHugh Eq. 1). Such equations are known as cable equations, because of their similarity to cable or transmission line equations [25], and the corresponding models of nerve conduction are known as cable models.

Cable models are especially suited to predict outcomes of biological experiments that measure axonal conduction velocity because they can be used to simulate wave propagation through successive nodes. Goldman and Albus [16] described a cable model and focused on characterizing the relationship between conduction velocity and fiber diameter in myelinated axons, which was found to be nearly linear for realistic axon models. Over the next decade this model was further studied by several groups using alternative methods for numerical integration and slightly differing parameter values and to obtain myelinated conductions velocities between 12 and 22 m/sec and realistic dependence of conduction velocity upon fiber diameter [17], [18].

Blight [19] studied a similar resistance-capacitance network representing a chain of 20 internodes. In the equivalent electrical circuit, internodes l-9 and 1 l-20 were each represented by a single segment, containing the resistance and capacitances of the internodal axolemma and myelin sheath, separated from the nodal circuits on either side by the resistance of the axon core. Internode number 10 was broken into 10 shorter sub-segments such that the importance of paranodal parameters could then be explored. The model quantitatively reproduced the voltage response of the axon to injected current pulses and to propagated action potentials, using Frankenhaeuser-Huxley kinetics. Blight highlighted the importance of the input resistance of the internode and the storage of charge at the axolemma. Later Stephanova and Bostock [20], [21] created a distributed-parameter model of the myelinated human motor nerve fiber, and also highlighted the role of paranodal resistance. Similarly, McIntyre and coworkers [22] specifically included paranodal resistance in their model and studied the biophysical mechanisms underlying changes in excitability following an action potential. The re-derived cable equations of Nygren and Halter [24] have sodium channels that are localized at the node, whereas potassium channels and the transmembrane sodium/potassium pump are located predominantly away from the node. In the present study we have retained the concept of separation of sodium and potassium channels, now well established as a constant feature of myelinated axons, and we have added the capability to systematically adjust parameters related to subtle injury during neurotrauma.

Our model is obviously limited in being a theoretical rather than an experimental study in which only selected features of the complete complex biological system are included. Ours is a deterministic rather than a stochastic model, hence average or typical values of parameters are used and statistical variation in axon diameter, axon spacing or internodal width, density and distribution of ion channels, thickness of the myelin sheath, and so on are ignored for the sake of answering larger questions about an idealized or typical myelinated axon. Noteworthy is the assumption that internodal myelin is an ideal insulator relative to uninsulated nodes. We ignore internodal leakage currents, which would likely change nodal currents and voltages by only a few percent.

### Conclusions

The straightforward cable model presented here recapitulates how the arrangement of myelin covered segments of axons, punctuated by narrow bare nodes of Ranvier, can produce saltatory conduction. The function of nodes of Ranvier in speeding impulse conduction is related to the ultrastructure of the nodes themselves. Normal myelinated nerve conduction velocity is inversely related to the node width, s, and to the nodal ratio, s/d, in the presence of intact paranodal resistance, R_p_. The narrow axial width of the nodes of Ranvier is the key to fast conduction. The biological tradeoff is that even a small mechanical retraction of myelin from very narrow nodes can cause large changes in conduction velocity. Very narrow nodes would be very fast, but exceedingly delicate. Hence evolution may have already found a near optimal tradeoff between quickness and toughness.

Modeling also suggests how nodes can malfunction in disease. Subtle mechanical, chemical, or immunological injury to the nodes of Ranvier, causing small increases in effective node width, s, and nodal capacitance, C, which could be easily overlooked using ordinary light microscopes, can contribute to pathology of conditions such as spinal cord injury and multiple sclerosis that cause retraction of myelin. The effects of node stretching are magnified by detachment of paranodal myelin, a process that decreases paranodal resistance and increases repolarizing potassium current above the normal level. Thus the nodes of Ranvier are highly effective but somewhat fragile devices for increasing nerve conduction velocity and decreasing reaction time in vertebrate animals. Increased nodal capacitance or decreased paranodal resistance caused by subtle myelin retraction and detachment cause slowed saltatory conduction and ultimately conduction block. Better understanding of the pathophysiology of nodal injury may lead to new treatments as well as heightened awareness of the dangers of subtle neurotrauma in closed head injury.

## Supporting Information

Figure S1 **Electrical model for one side of one node of Ranvier with potassium conducting regions separated from the node by high resistance paranodal regions.**
(TIFF)

Appendix S1
**Electrical effects of segregated ion channels.** Detailed treatment of the additional complexity of ion channel segregation in the nodal, paranodal, and juxtaparanodal regions, leading to quantitative estimation of normal paranodal resistance 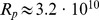 ohms.(DOC)
