# Supplementary material for: Subtle Paranodal Injury Slows Impulse Conduction in a Mathematical Model of Myelinated Axons
Source: PLoS One. 2013 Jul 3;8(7):e67767. doi: 10.1371/journal.pone.0067767 (PMC3701069; doi:10.1371/journal.pone.0067767)
Supplement: Appendix S1 — Electrical effects of segregated ion channels. Detailed treatment of the additional complexity of ion channel segregation in the nodal, paranodal, and juxtaparanodal regions, leading to quantitative estimation of normal paranodal resistance ohms. (DOC) [file pone.0067767.s002.doc]

**Appendix. Electrical effects of segregated ion channels.**

Figure S1 illustrates the additional complexity of ion channel segregation in the nodal, paranodal, and juxtaparanodal regions. It is now known that the sodium channels, carrying inward sodium current, are concentrated in the bare nodal membrane, represented in Figure S1 by capacitance, C_n_. Potassium channels, carrying outward repolarizing current are located in the juxtaparanodal regions on either side of the node. The nodal and juxtaparanodal regions are separated by the paranodal region, where tight cell-cell attachments of myelin membranes to the axonal membrane occur, isolating C_n_ from the source of potassium current. High paranodal resistance is represented by R_p_, which is a lumped value for juxtaparanodal regions on both sides of the node, as is current source i_K_. The axoplasmic resistance between the node and juxtaparanodal regions is considered negligible in comparison with R_p_. Let electrical potential in the juxtaparanodal extracellular space be V_o_ and the intra-axoplasmic potential be V_i_. The zero-voltage reference potential is that of extracellular fluid outside the node, as shown in Fig S1.

For juxtaparanodal membrane area A_K_, potassium equilibrium potential, E_K_ and potassium conductance per unit area, G_K_, the potassium current is given by

$i_{K}=G_{K}A_{K}\left((V_{i}-V_{o})-E_{K}\right)$, where (1)

$V_{o}=i_{K}R_{p}$. (2)

Equations (1) and (2) can be solved together for i_K_ to give

$i_{K}=\frac{V_{i}-E_{K}}{ \frac{1}{A_{K}G_{K}}+R_{p} }$ , (3)

which corresponds to Equation (3b) in the main text.

To estimate the numerical value of R_p_ in this model for use in computing specific numerical results, we assume that specific juxtaparanodal potassium conductance per square centimeter is similar to classical textbook values (peak value 0.013 S/cm^2^), that current is limited by R_p_, and that the duration of increased potassium conductance during the action potential, Δt_2_, is about 1 msec. Also the resting nodal charge

$Q=\int i_{Na}dt\cong\int i_{K}dt\approx\frac{E_{m}}{2}\cdot\frac{\Delta t_{2}}{R_{p}}$, (4)

where E_m_ is the resting transmembrane potential, and we estimate the mean transmembrane potential in the time domain during repolarization (when potassium channels are open) as one half E_m_. The resting nodal charge, can also be expressed in terms of E_m_, the bare nodal area, and the specific membrane capacitance, C_m_, per square centimeter as

$Q=C_{m}2\pi r_{a}s E_{m}$ . (5)

Combining (4) and (5) we obtain,

$R_{p}\approx\frac{\Delta t_{2}}{4\pi r_{a}s C_{m}}$.

Estimating for the normal node of Ranvier C_m_ = 1 x 10^-6^ Farad/cm^2^, Δt_2_ = 0.001 sec, r_a_ = 5 x 10^-4^ cm, and s = 5 x 10^-4^ cm, with 1 Farad = 1 sec/ohm,

$R_{p}\approx3.2 \cdot10^{10}$ ohms.

The fact that the model reproduces normal saltatory conduction with normal membrane repolarization confirms the reasonableness of the twin assumptions that juxtaparanodal potassium conductance is similar to that for typical non-myelinated nerve cell membranes and that paranodal resistance is a dominant current limiting factor. Otherwise, it would be necessary to assume unrealistically that paranodal resistance is negligible and that potassium channel density in the juxtaparanodal region is unusually low compared to sodium channel density. This alternative assumption would conflict with immunohistochemical results and also with results showing that the potassium channel blocker, 4-aminopyridine, can restore conduction in injured myelinated axons.
